# The engine initiating tissue regeneration: does a common mechanism exist during evolution?

**DOI:** 10.1186/s13619-020-00073-1

**Published:** 2021-04-05

**Authors:** Yanmei Liu, Wilson Pak-Kin Lou, Ji-Feng Fei

**Affiliations:** 1 Key Laboratory of Brain, Cognition and Education Sciences, Ministry of Education; Institute for Brain Research and Rehabilitation, South China Normal University, 510631 Guangzhou, China; 2 School of Life Sciences, South China Normal University, 510631 Guangzhou, China; 3 Research Institute of Molecular Pathology (IMP), Vienna Biocenter (VBC), Vienna, Austria; 4 Guangdong Provincial People’s Hospital, Guangdong Academy of Medical Sciences, 510080 Guangzhou, China

**Keywords:** Tissue regeneration, ROS, Immune response, Nerve factors, Cell types, Regeneration initiating factors, Injury

## Abstract

A successful tissue regeneration is a very complex process that requires a precise coordination of many molecular, cellular and physiological events. One of the critical steps is to convert the injury signals into regeneration signals to initiate tissue regeneration. Although many efforts have been made to investigate the mechanisms triggering tissue regeneration, the fundamental questions remain unresolved. One of the major obstacles is that the injury and the initiation of regeneration are two highly coupled processes and hard to separate from one another. In this article, we review the major events occurring at the early injury/regeneration stage in a range of species, and discuss the possible common mechanisms during initiation of tissue regeneration.

## Background

**Fig. 1:**
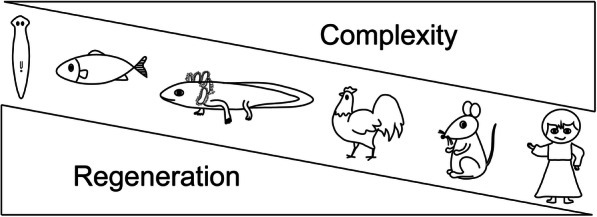
The regenerative capability of organisms gradually decreases over the course of  evolution, concomitant with the increase in complexity of the organism. From left to right: Turbellaria (planarians), fish (zebrafish), amphibians (axolotls), rodents (mice), primates (humans)

Regenerating damaged tissue/organs is highly clinically relevant. However, mammals, including humans, have only very limited capability for regeneration. Comprehensive understanding of the principles of regeneration will give insights to develop possible regenerative therapies. To this end, studying mammalian development may provide critical hints for regeneration, since regeneration is very similar to the developmental process in a number of systems (Nacu and Tanaka 2011). Another valuable approach is to use regenerative animal models to study naturally occurring regeneration processes. During evolution, a variety of species, including invertebrates (such as hydra, planarians, worms and insects) and vertebrates (such as fish, frogs and salamanders) exhibit great regenerative abilities and are used as model organisms in the field of regenerative biology (Fig. 1) (Tanaka and Reddien 2011; Gemberling et al. 2013; Shi et al. 2015).

Upon tissue injury, a sophisticated cascade of chain reactions is triggered, which finally either leads to successful regeneration, partial tissue repair or just wound healing (failed regeneration) (Brockes et al. 2001; Tanaka 2016). Both the mechanisms induced specifically by injury and the machineries employed during development are necessarily recruited for successful regeneration to occur (Nacu and Tanaka 2011; Roensch et al. 2013). The entire regeneration programme could be roughly categorized into the following major steps: Firstly, the cells adjacent to the lesion rapidly respond to the damage and produce wound signals. Secondly, the wound signals, or the downstream regeneration signals triggered by them, activate progenitor cells to proliferate. In vertebrates, depending on animal species and organs, the lineage restricted progenitor cells may be pre-existing (McHedlishvili et al. 2007; Kragl et al. 2009; Tu and Johnson 2011), or may be derived from trans-differentiation (Tsonis and Del Rio-Tsonis 2004; Barbosa-Sabanero et al. 2012), or de-differentiation/rejuvenation of (terminally) differentiated cells (Knopf et al. 2011; Tu and Johnson 2011; Rodrigo Albors et al. 2015; Wang et al. 2015; Gerber et al. 2018). However, in invertebrates such as planarians, pluripotent neoblasts are activated during regeneration (Zeng et al. 2018). Thirdly, newly born lineage specific progenitor cells or neoblasts differentiate into diverse cell types to reconstitute the lost tissue. Lastly, the regenerating tissue ceases to grow further after it has reached the correct size. Coordination of each step has to be precisely controlled. Misregulation of any single step would lead to regeneration failure.

Although many exciting advances have been made in the field of regenerative biology over the past years, many fundamental questions remain unresolved, such as how are the initiation and termination of tissue regeneration precisely determined? And how is the correct regeneration response triggered by different types of injury? Regarding the origins of the signals to initiate regeneration, whether it is successful tissue regeneration or scar formation, the downstream response is always triggered by tissue damage and subsequent wound signaling. Therefore, different hypotheses could be proposed. One possibility is that the injury-triggered signals such as apoptosis and ROS (reactive oxygen species) directly stimulate regeneration (Bergmann and Steller 2010; Love et al. 2013; Mescher et al. 2017). Another possibility is that the wound signals need to be converted into a different form, such as immune- or nerve-signaling (Fogarty et al. 2016; Santabarbara-Ruiz et al. 2019; Arenas Gomez et al. 2020) to trigger tissue regeneration. However, the injury response and initiation of tissue regeneration are highly coupled events, which creates difficulty to tease the two processes apart and identify the exact regeneration signals.

**Fig. 2:**
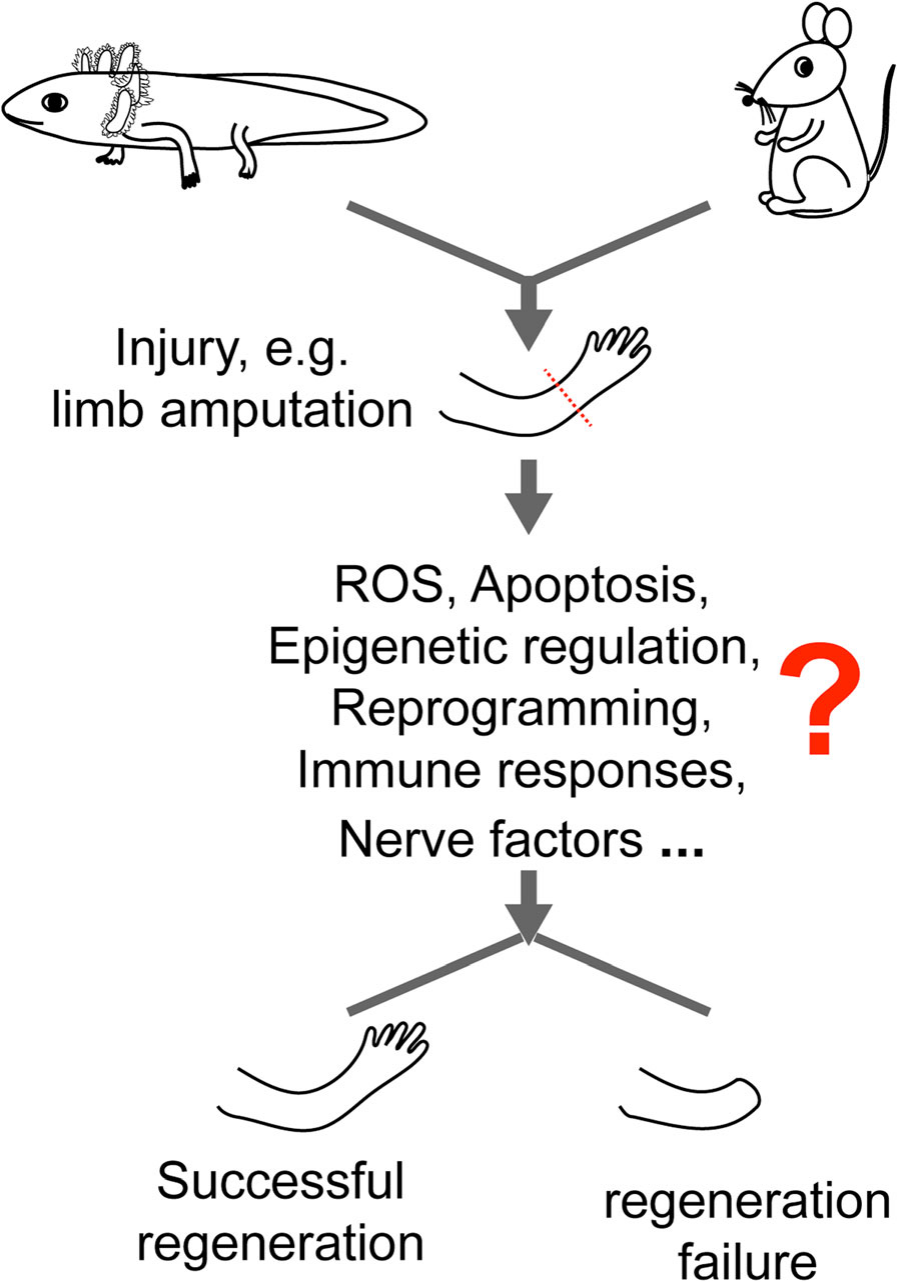
The early wound responses occurring in typical regenerative (axolotls) and non-regenerative (mice) species after injury

In this review, we summarize the findings reported from a range of organisms, with particular focus on the major responses occurring immediately after tissue damage or at early stages of regeneration, including calcium (Ca^2+^) signaling, ROS, apoptosis, inflammation (immune-response) and nerve-related factors (Fig. 2), cellular responses and the epigenetic regulation of regeneration. These aspects may be directly or indirectly involved in the initiation of tissue regeneration. In terms of evolution, we further discuss the potential conservative mechanisms that initiate tissue regeneration.

## Main Text

### Ca^2+^ signaling - the initial trigger of tissue regeneration?

As one of the most universal second messengers, Ca^2+^ plays a critical role in many biological processes. Ca^2+^ transiently transduce signals by regulating protein activity. Chronical Ca^2+^ signaling could also determine the cell identity by affecting the whole transcription program. The role of Ca^2+^ in regeneration has gained attention in many organisms including *C. elegans*, Drosophila, Xenopus and zebrafish. In *C. elegans*, both epidermal wounding and neural injury evoked Ca^2+^ entry which was further amplified by intracellular Ca^2+^ release. The injury-evoked Ca^2+^ signal is required for wound closure and axonal regrowth via triggering actin polymerization or activating Dual-Leucine-zipper-bearing Kinase-1 (DLK-1) respectively (Ghosh-Roy et al. 2010; Yan and Jin 2012; Sun et al. 2014; Xu and Chisholm 2014). Drosophila wing imaginal discs displayed slow, long-range intercellular Ca^2+^ waves in response to mechanical stress. Knockdown of the genes such as *Inositol-3-phosphate receptor* and *Innexin2*, which are required for the formation and propagation of these Ca^2+^ waves, impaired wing disc recovery after injury (Restrepo and Basler 2016). In Xenopus larva with amputated tails, Ca^2+^ transients were found to manifest in the regenerating muscle cells depending on Ca^2+^ release from ryanodine receptor-operated stores. Blockade of these transients prevented the activation and proliferation of muscle satellite cells and disturbed muscle regeneration (Tu and Borodinsky 2014). Using zebrafish larval tail fins as a model, wounding also induced very rapid and transient Ca^2+^ flashes in the epithelia. These Ca^2+^ transients were released from internal stores and required for fin regeneration (Yoo et al. 2012; Kujawski et al. 2014).

From an evolutionary point of view, Ca^2+^ is one of the earliest wound-induced signals, which can further induce ROS signaling in multiple species (described below) (Niethammer et al. 2009; Xu and Chisholm 2014; Fu et al. 2020). It may evolutionarily function as one of the primary triggers to initiate tissue regeneration upon injury. However, how Ca^2+^ signaling regulates progenitor cell behavior or reprograms previously differentiated cells during regeneration remains to be answered.

### ROS plays essential roles in tissue regeneration

Production of ROS, particularly hydrogen peroxide (H_2_O_2_), is rapidly induced after wounding and is required for tissue regeneration in a diverse range of species, from invertebrates (e.g. Drosophila, *C. elegans*, hydra), low vertebrates (e.g. zebrafish, frogs, salamanders) to high vertebrates (mammals) (Love et al. 2013; Xu and Chisholm 2014; LeBert et al. 2018; Romero et al. 2018; AL Haj Baddar et al. 2019; Santabarbara-Ruiz et al. 2019). H_2_O_2_ synthesis in zebrafish that is mediated by the enzyme dual oxidase (DUOX) (Niethammer et al. 2009), or locally produced in mitochondria in *C. elegans* (Niethammer et al. 2009; Xu and Chisholm 2014), are likely triggered by injury-induced Ca^2+^ influx. The release of ATP, another early signal after tissue damage, also stimulates H_2_O_2_ production by DUOX (de Oliveira et al. 2014). The H_2_O_2_ gradient generated in the regenerating tissue is detected by the redox-sensitive Src family kinase Lyn in leukocytes and mediates initial neutrophil recruitment to the wound (Yoo et al. 2011). To prevent excessive tissue damage, myeloperoxidase delivered by neutrophils removes H_2_O_2_ rapidly after injury (Mathias et al. 2006).

The importance of ROS signaling is highlighted by studies in multiple species, in which tissue regeneration is inhibited when injury-induced production of ROS is blocked, via genetic approaches or pharmacological treatments (Love et al. 2013; Labit et al. 2018). Recent studies using Drosophila wing imaginal discs as a model have provided additional mechanistic insight into the function of ROS in regeneration. Upon injury, cells proximal to the injury site receive high levels of ROS, which activates the Apoptosis signal-regulation kinase 1 (Ask1) to promote apoptosis (Santabarbara-Ruiz et al. 2019). The neighboring cells receiving lower levels of ROS have increased levels of activated Akt, a kinase downstream from the insulin pathway, which attenuates Ask1 activity via phosphorylation. The attenuated Ask1 activity leads to moderate levels of *c-Jun N-terminal kinase* (*JNK*) signalling and activation of *p38* signaling, both of which triggers a regenerative response in the surviving cells (Santabarbara-Ruiz et al. 2019). In another study, a positive feedback loop of ROS production was reported in the regenerating tissue. The gene *Moladietz*, which encodes DUOX-maturation factor *NIP*, was upregulated by ROS-induced *JNK* signaling. *NIP* in turn induces ROS production, so that *JNK* signaling in the regenerating tissue is maintained to ensure maximal tissue regrowth (Khan et al. 2017).

ROS was also reported to regulate voltage-gated sodium channels to initiate early bioelectric activities required for regeneration (Ferreira et al. 2016). Another identified ROS downstream pathway essential for regeneration is *Wnt/β-catenin* signaling and its major downstream targets fibroblast growth factor (FGF) 20 (Love et al. 2013).

### The role of apoptosis in tissue regeneration

Following injury, damaged cells adjacent to the lesion site will undergo apoptosis, a mechanism to remove irreparable cells. Many studies show that apoptosis-induced compensatory proliferation plays an essential role in tissue homeostasis of multiple organisms, such as drosophila imaginal disc and small intestine (Jiang et al. 2009), as well as zebrafish skin epithelial tissue (Brock et al. 2019). It appears that apoptosis may play similar roles in the context of tissue regeneration. Upon injury, it has been reported that apoptotic cells can produce Wnt or JNK signaling molecules to induce compensatory cell proliferation during regeneration (Ryoo et al. 2004; Chera et al. 2009; Jiang et al. 2009). In contrast, inhibition of apoptosis via disrupting caspase activity could block tissue regeneration in drosophila and hydra (Ryoo et al. 2004; Chera et al. 2009). Similar phenotypes were also documented in several other models of regeneration across different species, including planarians, newts, Xenopus, and mammals (Hwang et al. 2004; Vlaskalin et al. 2004; Tseng et al. 2007; Li et al. 2010; Pellettieri et al. 2010; Gauron et al. 2013), as reviewed previously (Bergmann and Steller 2010; Fogarty et al. 2016; Diwanji and Bergmann 2018). Interestingly, studies in zebrafish fin regeneration revealed that there are two waves of apoptosis after injury. The peak of the first wave appears rapidly at about 1-h post injury, and the second wave peaks at 15–18 h after injury. The second apoptosis peak is at least partially induced by the pro-regenerative ROS signal, and it is specific to fin regeneration, because a mere wounding on the fin only induce the first, but not the second wave of apoptosis. Both blastema formation and regeneration are impaired when the second apoptosis is chemically inhibited (Gauron et al. 2013). Considering the injury-induced activation of ROS signaling and apoptosis occurs for nearly all species, irrelevant to their regenerative capability, it may be worthwhile to systematically investigate whether the mode of ROS production and apoptosis behave differently between regenerative and non-regenerative species.

Furthermore, apoptosis may function as one intrinsic factor involved in tissue regeneration, as part of the cell fate reprogramming machinery. Heng and colleagues have shown that upon newt limb amputation, post-mitotic multinucleated muscle cells undergo massive apoptosis. A proportion of mononucleated cells generated in this process do not follow through with cell death, and are instead reprogrammed into proliferative myoblasts and take part in regeneration (Wang et al. 2015).

### The role of immune responses in tissue regeneration

The immune system plays an essential role in tissue regeneration and homeostasis. Inflammation response activates rapidly after the injury to recruit neutrophils, monocytes, and other innate immune cells to clear cell debris and remove invaded microbes. Compelling evidence points out that precisely regulated inflammation is critical for regenerative competence. Dampening inflammation with immunosuppressive glucocorticoids at the time of amputation impairs blastema formation and limits regeneration in zebrafish and Xenopus (Mathew et al. 2007; King et al. 2012). On the other hand, induction of persistent inflammation with Beryllium (Be^2+^) inhibits limb regeneration in both salamander and Xenopus (Thornton 1949; King et al. 2012). The inflammatory response is necessary to initiate repair and regeneration, in particular for blastema formation and new tissue patterning (Mescher et al. 2013). By secreting chemokines and other inflammatory mediators, macrophages are the key immune cells controlling the inflammatory status. They can be either tissue-resident macrophages or monocyte-derived macrophages recruited from blood after injury. By sensing and responding to environmental signals, macrophages are polarized to “pro-inflammatory” M1 macrophages or “anti-inflammatory” M2 macrophages at different stages during repair and regeneration (Mescher et al. 2017). However, classification of macrophages at different functional states is more complicated in vivo. For example, in the axolotls, inflammatory and anti-inflammatory markers are simultaneously induced within the first 24 h after limb amputation. Depletion of macrophages leads to failure of limb regeneration, which can be restored by endogenous macrophage replenishment (Godwin et al. 2013). These results demonstrate that macrophages in axolotls are involved in establishment of a regeneration-permissive environment.

The classical role of M1 macrophages is to phagocytize cellular debris which not only creates space for new regenerated tissue, but also further activates the signaling cascade required for regeneration. For example, during liver regeneration, macrophages scavenging hepatocyte debris expresses Wnt3a, which then promotes differentiation of nearby hepatic progenitor cells to hepatocytes through activating *Wnt* signaling (Boulter et al. 2012). When the pro-inflammatory response subsides, macrophages produce numerous growth factors such as Platelet Derived Growth Factor (PDGF), Insulin-like growth factors (IGFs) and Transforming Growth Factor (TGF)-β to regulate progenitor cell proliferation and differentiation (Wynn and Vannella 2016). During limb regeneration in salamander, macrophages promote cell dedifferentiation to form the progenitor cell pool (Yokoyama 2008).

Angiogenesis and vascular remodeling are key components of tissue regeneration. Both M1 and M2 macrophages promote angiogenesis by secreting trophic factors, cytokines, proteases and Wnt ligands (Leor et al. 2016). Another possible pro-angiogenic mechanism has been reported that macrophages are able to transdifferentiate to endothelial progenitors or endothelial-like cells (Fernandez Pujol et al. 2000).

Macrophages also regulate synthesis of extracellular matrix (ECM) components required for efficient regeneration by secreting cytokines and soluble mediators to act on fibroblasts (Godwin and Rosenthal 2014). As the major source of ECM, fibroblasts can produce either a fibrotic scar or the ECM of regenerating tissue (Godwin and Rosenthal 2014). Macrophages also secret matrix metalloproteinases to degrade the collagen of damaged tissues, triggering remodeling of the ECM (Yokoyama 2008). During the repair process, macrophages produce ECM components including Collagen type I, α1 (Col1α1) and Resistin-like molecule α (RELMα) after integrating various signals from specific cytokines and local cues (Bouchery and Harris 2017). Specific signals from different organs may determine the tissue regenerative capacity, which is remarkably variable in mammals.

Thereafter, macrophages mainly exhibit anti-inflammatory effects and modulate the local inflammatory microenvironment to regulate regeneration (Ramachandran et al. 2015). IL-10, produced by regulatory T (Treg) cells, Th2 cells and macrophages play a critical role in polarization of macrophages to promote tissue regeneration (Saraiva and O’Garra 2010). In response to interleukin-10 (IL-10) and other inhibitory mediators, M2 macrophages further suppress inflammation by secreting a variety of anti-inflammatory mediators including IL-10 and TGF-β1 (Khalil et al. 1989; Said et al. 2010; Shouval et al. 2014). M2 macrophages also regulates IL-10- and TGF- β1-producing Treg cell differentiation (Soroosh et al. 2013), implicating an interplay of adaptive and innate immune cells in the resolution of inflammatory responses during regeneration.

Subsets of Treg cells have been reported to play important roles in muscle regeneration. These cells regulate macrophage polarization into a pro-regenerative state (Tidball and Villalta 2010), but restrict the infiltration of conventional T cells (Burzyn et al. 2013). Muscle Treg cells express the growth factor Amphiregulin that could directly enhance satellite cell differentiation and improve muscle repair.

From a evolutionary point of view, evolution of an advanced adaptive immune system corelates with a loss of regenerative ability. Primitive animals with greater regeneration abilities only possess innate immunity. Whereas more evolved vertebrates, which possess the more complex and advanced adaptive immune system, retain very limited regeneration ability. Xenopus gradually lose their regenerative ability after the peak of metamorphosis when the immune system is fully developed. Salamanders possessing regenerative ability throughout the whole life have strong innate immune system but likely lack key adaptive immune responses. Therefore, it could be speculated that an advanced adaptive immune system may have some inhibitory effects on regeneration.

### Nerves and nerve-related factors---the central player of tissue regeneration?

Another critical aspects that have a fundamental impact on tissue regeneration are nerves and nerve-related factors. Nearly two-hundred years ago, nerve-dependent regeneration was first described during limb regeneration in a salamander species (Todd 1823). Either in a larval or adult urodele, denervation of limb nerves led to an inhibition of blastema formation, but not wound healing. Upon re-innervation, i.e. the re-growing of the limb nerve back to the injury site, blastema formation and limb regeneration were fully restored (Butler and Schotte 1941; Schotte and Butler 1941; Singer and Egloff 1949). These findings, together with the follow-up intensive studies from Singer and colleagues, demonstrated the chemical nature of nerve-dependent limb regeneration, which led to the proposal of the neurotrophic hypothesis: within a given area, the number of axons, and therefore the associated neurotrophic factors, must reach a certain threshold for regeneration to occur (Singer 1952; Singer 1964; Zika and Singer 1965). In past decades, many such kinds of neurotrophic factors, secreted from injured nerves or Schwann cells and playing essential roles in nerve-dependent regeneration, have been identified, including bone morphogenetic proteins (BMPs) (Satoh et al. 2010; Makanae et al. 2016), FGFs (Mullen et al. 1996; Han et al. 2001), keratinocyte growth factor (KGF, FGF7) (Satoh et al. 2008), Substance P (Satoh et al. 2008), newt anterior gradient (nAG) (Kumar et al. 2007), Neuregulin-1 (Farkas et al. 2016), and so on, as previously reviewed (Nye et al. 2003; Mitogawa et al. 2014; Satoh et al. 2015; Satoh et al. 2016; Satoh et al. 2018).

Until now, the phenomenon of nerve dependency of tissue regeneration has been observed in a broad range of species. In invertebrates, starfish arm regeneration relies on the presence of the radial nerve located at the amputation plane. Destroying the connection between the amputation plane and the central ring nerve blocked regeneration, which is similar to the denervated limb regeneration defects in salamanders (Huet 1975). However, even in the most classical regenerative invertebrate species like hydra and planarians, it is still not completely clear about the role of nerves in regeneration. In vertebrates, in addition to salamanders, peripheral nerves also play essential roles in regeneration of lower vertebrates such as fin and heart regeneration in zebrafish (Simoes et al. 2014; Mahmoud et al. 2015), and limb regeneration in Xenopus (Suzuki et al. 2005). Florescent tracking of nerve FGF and BMP provided direct evidences that these factors are transported through the long axons to the injury sites and support the appendage, such as limb regeneration (Satoh et al. 2016).

The tissue regeneration ability of mammals is in general very lacking (Fig. 1). In particular, the regeneration ability is significantly reduced from early development to adulthood in mammals. Studies of heart regeneration in newborn mice have revealed that nerves are involved in tissue regeneration. Pharmacological blocking of nerve function inhibits heart regeneration in newborn mice, but the regeneration defects could be rescued by providing neurotropic factors Neuregulin 1 or nerve growth factor (Mahmoud et al. 2015). This is similar to what has been observed in salamanders. It is very difficult to study nerve-dependency of tissue regeneration in adult mammals, due to the general lack of regeneration capability in most tissue/organs. Clark and colleagues discovered that Murphy Roths Large (MRL) mouse were able to regenerate their damaged tissue very well, for example without fibrotic scarring in an ear punch-hole injury model (Clarke et al. 1988), and this has been confirmed by many other research groups (Balu et al. 2009; Buhimschi et al. 2010; Gawriluk et al. 2016). Using the ear punch-hole model, it has been shown that the regeneration of cartilage and epithelial structures is nerve-dependent. The proximal end of the hole (close to the ear base) regenerates faster and produces the majority of cell mass for the blastema, when compared to the distal end (close to the ear tip). This difference is correlated to the amount of local nerve supply. There are more axons of the auricular nerve that invades the ear tissue via the ear base, located at the proximal end (Buckley et al. 2012). Denervation at the ear base via nerve transection severely impaired wound healing and regeneration (Buckley et al. 2012).

Is there a common molecular/cellular mechanism underlying such an evolutionarily conserved nerve-dependent tissue regeneration process? During newt limb regeneration, Kumar and colleagues have showed that nAG, a ligand secreted from Schwann cells, interacts with the cell surface molecule Prod 1 to promote the proliferation of blastema cells (Kumar et al. 2007). Remarkably, ectopic expression of nAG can nearly fully rescue the regeneration defects of denervated and amputated limbs (Kumar et al. 2007). nAG homologs are present in mammals such as mouse and human, but Prod 1 is specific to newts (Kumar et al. 2007). However, it is too early to say nAG-Prod 1 signaling is a key signaling pathway that distinguishes regenerative from non-regenerative phenomena, because it has not been identified so far in other regenerative species such as axolotls and planarians. It is likely that different mechanisms exist in different regenerative species.

Considering that regeneration capability generally decreases throughout evolution from cold- to warm-blooded animals, Korotkova and colleagues made a hypothesis that some unique regenerative factors may be specifically present in cold-blooded vertebrates, but have been lost in warm-blooded animals during evolution. The loss of genes encoding these factors in ancestors of warm-blooded species led to a general reduction of regenerative abilities (Korotkova et al. 2019). Taking advantage of bioinformatic screening, they found a gene, named *c-Answer*, which is expressed in the nervous system and regulates limb regeneration in Xenopus. c-Answer is a transmembrane protein that interacts with FGF receptor, one of the previously identified nerve-dependent regeneration factors, and promotes MAPK/ERK signaling (Korotkova et al. 2019).

A recent study from Lin’s lab has reported the nerve factor Melanocortin 4 receptor (Mc4r)/ α-MSH are required for both Xenopus tadpole limb and mouse digit tip regeneration (Zhang et al. 2018). Loss-of-function studies by morpholino treatment in Xenopus or knockout of *Mc4r* in mice, inhibited blastema formation, but not wound healing. Implantation of α-MSH-soaked beads close to the amputation plane enhanced Mc4r expression and rescued regeneration of denervated Xenopus limbs (Zhang et al. 2018). This study identified a novel neurotrophic factor Mc4r/α-MSH signaling involved in nerve-dependent tissue regeneration. Interestingly, since Mc4r/α-MSH signaling is present in both Xenopus and mice, it makes sense to consider that this pathway may be an evolutionarily conserved mechanism in nerve-dependent regeneration, and it is worthwhile to further investigate its role in other species.

### Apical Epidermal Cap (AEC) - a potential amplification center of regeneration signaling?

Upon urodele limb or tail amputation, epidermal cells rapidly migrate to and cover the wound surface. Nerve is not required for wound healing, but the expansion of the wound epidermis, which results in the formation of a multiple cell-layered cap structure––AEC, is nerve dependent (Satoh et al. 2008). In turn, the AEC interacts with surrounding peripheral nerves to further produce mitogens and promote the proliferation of underlying blastema cells (Trampusch 1964; Satoh et al. 2010). Both nerves and the AEC are required for limb blastema formation, based on the fact that either denervation of the amputated limb or interrupt of the AEC can inhibit normal blastema formation (Thornton 1960; Thornton and Steen 1962; Mescher 1976; Tassava and Garling 1979). Many key regenerative molecules, such as Msx2 (Carlson et al. 1998), Sp9 (Satoh et al. 2008) and Dlx-3 (Mullen et al. 1996), BMP2 (Satoh et al. 2010), FGF8 (Christensen and Tassava 2000; Han et al. 2001) and Mc4r (Zhang et al. 2018) have been shown to be expressed in the AEC and play important roles on blastema formation. Interestingly, there are evidence showing that many of these AEC factors are either initially secreted from peripheral nerves, before being expressed in the AEC, such as BMP, FGF and Mc4r (Makanae et al. 2016; Satoh et al. 2016; Zhang et al. 2018); or indirectly induced by different nerve factors, such as the induction of SP9 expression in the AEC by the nerve factor KGF (Satoh et al. 2008). The AEC factors in turn signals on the underlying blastema cells to promote their proliferation.

It seems that by certain mechanisms, nerve regenerative signals can be transformed into either the same types of molecules or different downstream factors in the AEC cells, which in turn amplifies the nerve signals and further facilitates the induction and maintenance of the blastema structure. From this point of view, one of the roles of the AEC is to act as a signal amplifier of nerve factors. However, the AEC is not necessarily present in all cases of successful tissue regeneration. This may be relevant to the size of injuries. The larger the injury, the more an amplifier domain is needed to provide sufficient regeneration signals. It may be that the nerve-signal amplification step is more relevant to the successful regeneration of larger injuries.

### Cell origin and hierarchy in tissue regeneration

When the injury-induced regeneration initiating signals reach the relevant cells, these cells will be activated and enter the cell cycle to proliferate, differentiate and give rise to new tissue to rebuild the lost tissue/organ. In classical regenerative organisms, such as planarians, flatworms or salamanders, the progenitor cells participating in tissue regeneration first propagate to form a blastema under the wound epidermis. There are two key questions: What are the sources of regenerative cells? How are these cells activated to participate in regeneration?

It is known that in planarians, neoblasts are the cells contributing to regeneration. Remarkably, a recent study from the Sánchez Alvarado lab showed that a single neoblast could perform a function similar to that of pluripotent germ cells, regenerating all cell types in the entire body of an irradiated animal (Zeng et al. 2018). However, there is no evidence to support the existence of pluripotent stem cells in the complex tissues of vertebrates, such as during limb regeneration of salamanders. Instead, different types of lineage restricted residential progenitors are activated to produce the relevant tissues (Kragl et al. 2009). In many cases, these progenitors undergo dedifferentiation or trans-differentiation before participating in regeneration (Poss et al. 2002; Kragl et al. 2009; Hirata et al. 2010; McHedlishvili et al. 2012; Gemberling et al. 2013; Sandoval-Guzman et al. 2014; Fei et al. 2017). The trans-differentiation phenomenon is well documented in iris pigment epithelial cells during lens regeneration in newts, a classical model used for studying tissue regeneration (Tsonis and Del Rio-Tsonis 2004; Barbosa-Sabanero et al. 2012). In addition, several studies indicated that fibroblast and neural progenitors, participating in axolotl limb and spinal cord regeneration respectively, undergo a “rejuvenation-type of de-differentiation” process, meaning that these progenitors are converted from an “aged status” back to an “embryonic-like status”, prior to entering the cell cycle and taking part in tissue regeneration (Rodrigo Albors et al. 2015; Gerber et al. 2018). These recent findings, together with other evidences (Tanaka 2016; Stocum 2017), support the concept that regeneration is the local re-initiation of developmental processes (Nacu and Tanaka 2011; Roensch et al. 2013). Overall, the presence of pluripotent vs. unipotent/multipotent stem/progenitor cells represents two different mechanisms in inveterate and vertebrate tissue regeneration.

In contrast, studies in poorly-regenerative vertebrates have revealed that tissue-specific adult stem/progenitors do exist in multiple tissues/organs, such as spinal cord, muscle and skin (Raff 2003; Wagers and Weissman 2004; Comai and Tajbakhsh 2014; Sabelstrom et al. 2014). However, upon tissue damage, all the relevant progenitors fail to respond correctly to produce the necessary progenies, resulting in the failure of regeneration (Meletis et al. 2008; Currie et al. 2019).

**Fig. 3:**
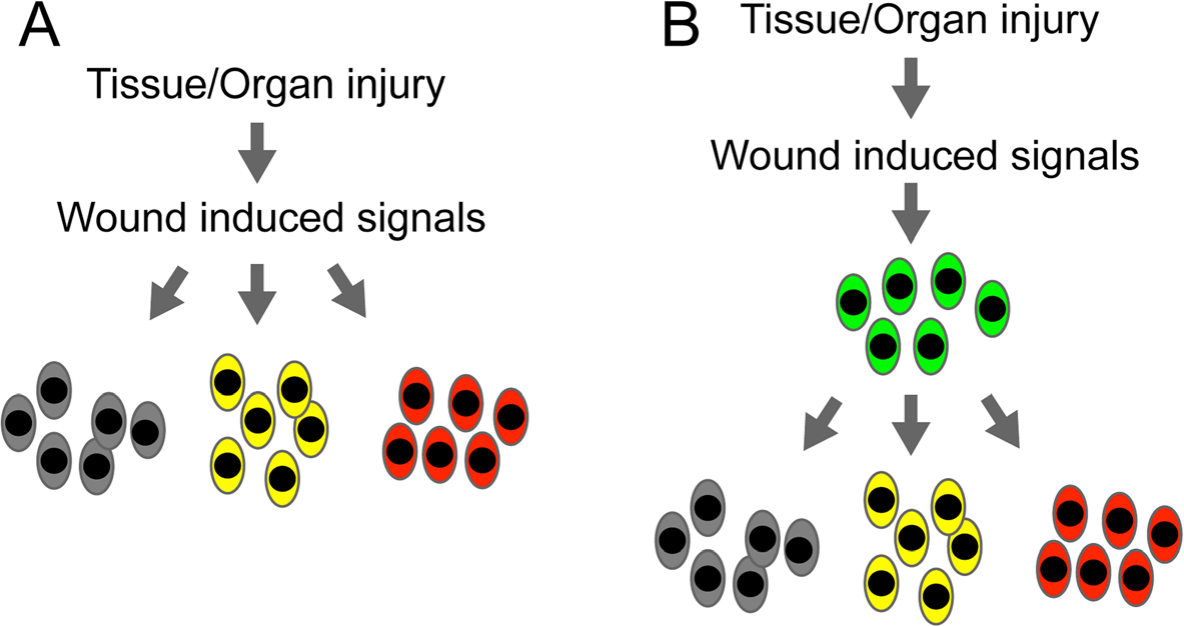
Two models of wound signal transduction pathways. The injury-induced wound signals either directly act on all progenitors as regenerative signals (**a**) or indirectly on the progenitors via an intermediate type of cells where the wound signals can be converted into regenerative signals

At the cellular level, since multiple types of cells are often involved in regeneration, is there a hierarchy in terms of the cells sensing or converting the injury-induced regenerative signals? Two possible models could be proposed to this question: 1) the wound-induced signals and the downstream induced regenerative signals that function on the progenitors to promote initiation of regeneration are accomplished in the same cells. In other words, the same type of cells sense the wound and produce regenerative signals (Fig. 3a). 2) The wound-induced signals are initiated in the cells sensing the injury, then transmitted to other types of cells, where they are converted into regenerative signals to stimulate various progenitors to form the blastema (Fig. 3b). Many studies demonstrated that given tissue/cell types do play dominant roles in the context of initiating tissue regeneration. Neurons or other relevant cells (for example cells in the AEC) are sufficient to induce blastema initiation and growth. It was found that supplementing identified nerve or AEC-derived factors alone could replace the function of nerves or the AEC and rescue the regeneration defects in the absence of either nerves or the AEC (Nye et al. 2003; Mitogawa et al. 2014; Satoh et al. 2016; Satoh et al. 2018; Stocum 2019). Remarkably, a recent work using Xenopus tail regeneration model identified a new type of regeneration-organizing cells (ROCs), which are located in the AEC and function on top of different progenitors to coordinate their proliferation. Removal of ROCs via surgical or genetic means inhibited regeneration, whereas regeneration capability could be restored in regeneration-incompetent tadpoles (e.g. stages 45–47) upon transplantation of ROC containing tissue to the amputation plane (Aztekin et al. 2019). It will be interesting further investigate whether ROC cells exist, in other regenerating frog tissues, or other regenerative organisms. In contrast, muscle tissue, including satellite cells, is dispensable for salamander limb regeneration. Knockout of *Pax3* gene in newts leads to the depletion of muscle stem cells and the loss of limb muscle during development. However, such muscle depleted limb can initiate regeneration properly (Elewa et al. 2017). In summary, all these collected evidences suggest that the presence of hierarchy in varied types of progenitor cells/tissues (nerve vs. muscle tissue) which do play different roles during the initiation of tissue regeneration.

### Epigenetic regulation of tissue regeneration---enhancers

It has been reported that epigenetic regulation is one of the earliest responses upon tissue injury. Enhancers are generally featured as open chromatin areas, interacting with certain transcription factors and bearing particular histone modifications, to regulate the expression of nearby genes (Long et al. 2016). Regeneration specific enhancers could be conserved during evolution and may play essential roles to activate regeneration at early stages (Darnet et al. 2019). By comparing the transcriptome profile from regenerating zebrafish heart and fin, Kang and collogues identified an enhancer sequence from the leptin b genomic locus, which could be activated rapidly in both zebrafish heart and fin blastema. Interestingly, this enhancer could also drive reporter gene expression in injured mouse heart tissue (Kang et al. 2016). This indicates that this enhancer may be conserved in multiple vertebrate species in evolution. In addition, during whole body regeneration of the acoel worm *Hofstenia miamia*, ATAC-seq analysis at the chromatin level revealed that early growth response (EGR) binding sites are prevalent injury-induced elements activated by binding to EGR proteins (Gehrke et al. 2019). Interestingly, EGR is one of the earliest genes activated after spinal cord injury in vertebrate axolotls (Rodrigo Albors et al. 2015), which suggests the role of EGR regulation in wounding/regeneration is possibly evolutionary conserved between invertebrates and vertebrates. Recently, taking advantage of comparative epigenetic analysis, the Sánchez Alvarado group investigated species-specific and evolutionarily-conserved cis-regulatory elements in regeneration using two related teleost fish, killifish and zebrafish (Wang et al. 2020). They identified several conserved regeneration-responsive enhancers (RREs), including known regeneration enhancer upstream of the inhibin beta A gene, and further found out that the presence of activator protein 1 (AP-1)-binding motifs is critical for a portion of identified RREs to function properly. Remarkably, both AP-1 and the AP-1 binding motif are present in mammals. However, the human AP-1 binding motif, when inserted into the killifish genome, did not respond in the same way as the fish AP-1 binding motif, which correlates to the decreased regeneration ability in mammals (Wang et al. 2020). This study suggests the regenerative functions of the RREs may be lost during evolution.

### Evolutionary perspectives

**Fig. 4:**
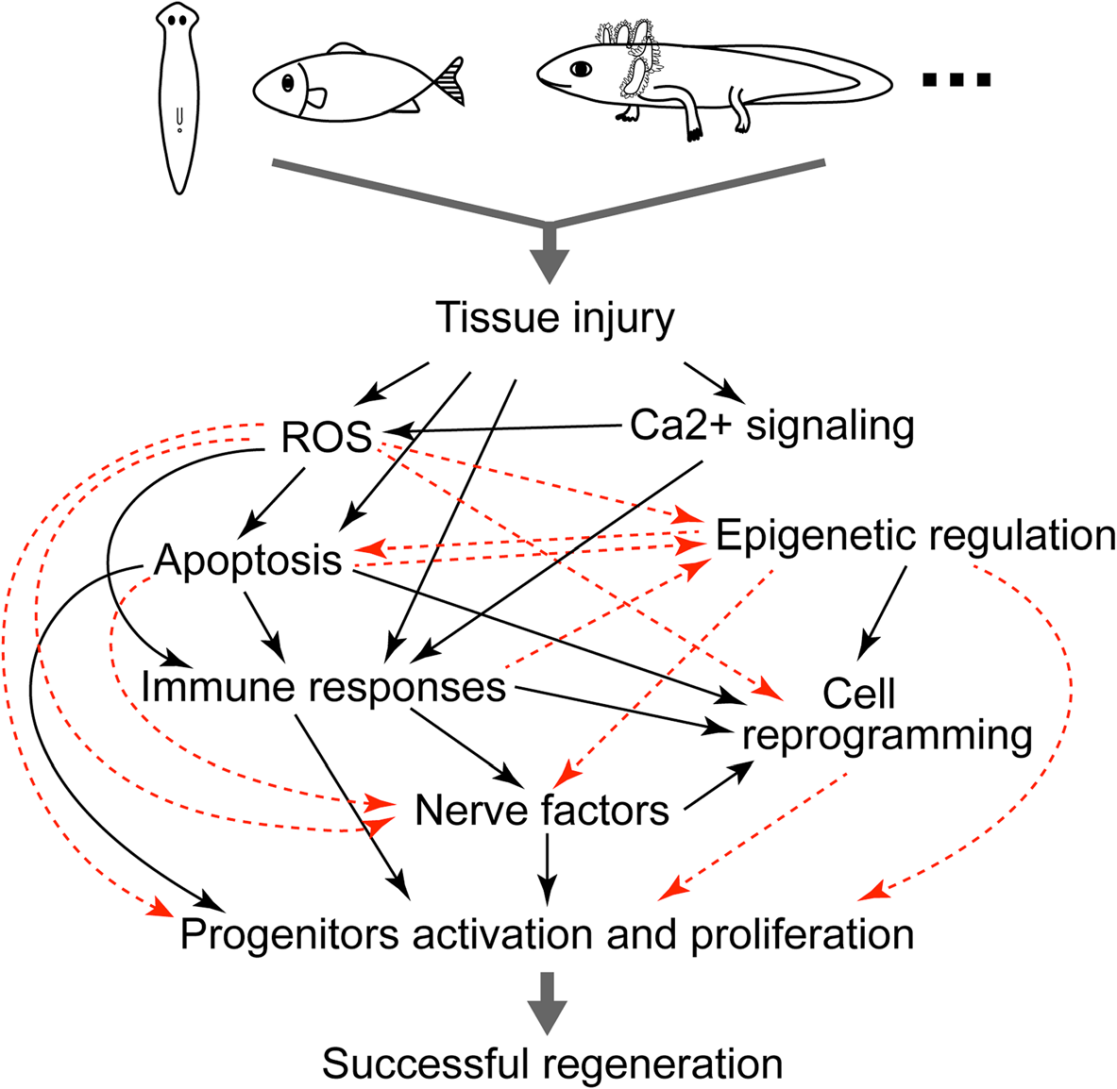
The interaction of tissue injury, the injury-induced early wound responses and progenitor activation, occurring immediately after injury or at the early stage of regeneration. Black solid arrows represent the confirmed interactions based on the studies from regenerative organisms; Red dashed arrows indicate potential interactions

From the moment of injury to successful regeneration, numerous molecular and cellular processes are involved. The common responses such as ROS, immune response, nerve dependency and epigenetic regulation were already reported in a broad range of species (Fig. 4). However, interactions between these major early responses are only partially revealed in some species (Fig. 4). This limited understanding makes it still difficult to determine what the exact signal for regeneration initiation is, let alone whether there is an evolutionarily conserved initiation mechanism.

There are many reasons for this. Firstly, tissue regeneration is a very complicated process involving many different cell types and signalling pathways. For simpler species, such as hydra or planarians, the exact molecular and cellular mechanisms of tissue regeneration are already poorly dissected. It is even more difficult to study reconstruction of complex tissues such as limbs in more complex species. From this point of view, it is important to focus on a simple regenerative species and comprehensively study its regeneration programme. Secondly, the regeneration phenomenon varies depending on damaged organs or species. In order to figure out the initial signals of tissue regeneration, it is necessary to systematically study the detailed molecular and cellular injury responses of different organisms, and to compare the differences between invertebrates and vertebrates, lower and higher vertebrates, and non-regenerative and regenerative species. Thirdly, injury response and regeneration are tightly coupled during regeneration. It is almost impossible to isolate and identify the exact signals that start tissue regeneration. Using the proper model may help to solve this issue. Accessory limb model (ALM) can induce an ectopic blastema that develops into a limb in the presence of skin lesions and nerve derivatives (Endo et al. 2004; Satoh et al. 2015; Nacu et al. 2016; Vieira et al. 2019). ALM converts an otherwise wound healing only response into a limb regeneration programme. And this could be harassed to study regeneration initiation mechanisms. Moreover, establishing new experimental systems to segregate wound healing and the onset of tissue regeneration will also be valuable. Furthermore, emerging new technologies, such as single cell sequencing and various “omics”, have already been applied to regenerative species such as axolotls and zebrafish (Gerber et al. 2018; Leigh et al. 2018; Hoang et al. 2020; Hou et al. 2020; Li et al. 2020), and will contribute to in-depth study of the current unresolved issues in the field of regeneration.
